# Perceptions of Family Medicine among first-year medical students at Aga Khan University, Nairobi, Kenya

**DOI:** 10.4102/phcfm.v18i1.5073

**Published:** 2026-02-17

**Authors:** Catherine Gathu, Lucy W. Mwangi, Peter Kioko, Tatiana Elwes

**Affiliations:** 1 Department of Family Medicine, Medical College, East Africa, The Aga Khan University, Nairobi, Kenya; 2 Research Division, Medical College, East Africa, The Aga Khan University, Nairobi, Kenya; 3 Amber Medical Clinic, Nairobi, Kenya; 4 Thistle Health Ltd, Nairobi, Kenya

**Keywords:** Family Medicine, medical education, medical students, medicine as a career, family physician

## Abstract

**Background:**

Family Medicine (FM) is vital in delivering comprehensive and continuous care essential for robust primary healthcare systems. However, it remains underappreciated in many settings, particularly in sub-Saharan Africa.

**Aim:**

This study aimed to assess the perceptions of FM among first-year medical students at Aga Khan University (AKU), Kenya.

**Setting:**

A cross-sectional survey was conducted between August 2024 and September 2024 among the first-year medical students at AKU. All were invited to participate via email.

**Methods:**

An 18-item questionnaire focused on three areas: perception and choice of medicine as a career, perception of FM and choice of FM as a career. Descriptive statistics were used to analyse the data collected.

**Results:**

Of the 59 first-year medical students, 49 participated (83% response rate). While 88% acknowledged FM’s importance, only 35% understood a family physician’s role. Only one participant indicated that they would choose FM as a career, while 61% were uncertain. Reasons for not considering FM included a lack of interest, limited knowledge and negative experiences with FM physicians. Only 16% reported that interactions with FM physicians and the first-year curriculum significantly improved their understanding of FM; many respondents disagreed that the first-year curriculum content advanced their understanding of FM.

**Conclusion:**

Integration of FM into undergraduate curricula could promote students’ interest. Additional research is needed to explore perceptions among medical students at different levels of their training and across different medical schools in our region.

**Contribution:**

This study establishes baseline perceptions of family medicine among first-year Kenyan medical students, identifying how knowledge gaps and limited curricular exposure undermine interest in the specialty.

## Introduction

According to the World Organization of National Colleges, Academies and Academic Associations of General Practitioners and Family Physicians (WONCA), Family Medicine (FM) physicians, also called family physicians, are expert doctors who are trained to provide comprehensive and ongoing primary healthcare to patients within the broader contexts of family, community and culture irrespective of age, gender or health condition.^1^ Since 1978, when PHC was defined,^2^ many countries have endeavoured to integrate primary healthcare (PHC) into their healthcare systems,^3^ helping to ensure the provision of holistic^4,5^ and cost-effective patient care.^6^ Ideally, family physicians lead the PHC teams within healthcare facilities.^7,8^ Over the years, FM training has been enhanced in several countries, including Kenya, where postgraduate training in FM commenced in 2005. Two years later, a nationwide FM strategy was released, augmenting the recognition and importance of this speciality for healthcare provision to individuals and communities.^8,9,10^

However, several challenges hinder the progress of FM as a specialty in Kenya. Among these is that FM remains ill-defined and poorly understood in the community and among other medical specialists and health policymakers.^7,8^ Furthermore, as PHC is generally under-resourced, funding is mostly allocated to curative care rather than preventive services.^7,8,11^ In addition, some family physicians find themselves addressing human resource shortages within the healthcare system rather than practising in the roles for which they were specifically trained.^7^ The summation of these challenges means that the training and production of family physicians are slow and their numbers few,^3,12,13^ leading to a limited pool of FM specialists available to practice and teach undergraduate and postgraduate medical trainees on the core FM concepts.^9,11^ Overall, the low number of practising FM specialists curtails the contact time and role modelling for medical trainees despite the consensus that early exposure to the concepts of FM and PHC in medical school is beneficial.^7,11^

Some African countries, including South Africa^14,15,16^ and Nigeria,^17^ have integrated FM into the curricula of undergraduate medical education. In Kenya, only one out of thirteen medical training schools has clinical FM exposure at the undergraduate level.^11^ In addition, there is a notable lack of research about the practice of FM and how it is perceived in Kenya.

In 2023, the Aga Khan University (AKU) in Nairobi, Kenya, launched an innovative 6-year, trimester model, Bachelor of Medicine and Bachelor of Surgery (MBChB) curriculum to train undergraduate students. This is structured into two phases, a 3-year preclinical phase followed by a 3-year clinical phase. Here, FM is not taught as a stand-alone course but is integrated in the curriculum through courses facilitated by FM physicians who serve as key educators across the programme. They teach consultation skills and emergency medicine in the early years of training and instruct on leadership, clinical governance and entrepreneurship during the clinical phase. This is done in tutoring small groups, leading interdisciplinary course modules and through mentorship. Medical students therefore receive early, albeit indirect, exposure to key FM concepts such as person-centred care, continuity of care and interprofessional teamwork.

There is a need to evaluate the effect of early exposure to FM among the undergraduate students, and the findings used to inform medical school curricula development and ultimately shape the landscape of PHC in Kenya. This study, therefore, set out to understand the perceptions among first-year medical students at AKU towards FM and assess the influence of the first-year curriculum on the choice of FM as a future career.

## Research methods and design

### Study design, setting and participants

A baseline mixed-methods survey to gain insight into the perception of FM among first-year Bachelor of Medicine and Bachelor of Surgery (MBChB) students at the Aga Khan University, Kenya, was conducted between August 2024 and September 2024. A total of 59 students had been enrolled into the programme. As this was a newly established programme at the time of the study, the first-year students were the only cohort included. The survey was conducted while the participants were in their final trimester of their first year, and all students were invited to participate via email.

### Survey development

The survey comprised 18 items adopted from a similar previously validated tool.^12^ The tool had five main parts: (1) Participant data, assessed by three items (age, gender and curriculum of high school attended); (2) perception and choice of medicine as a career assessed by four items; (3) perception of FM as a speciality assessed by five items; (4) ways of interaction of students and FM physicians assessed by one item; and (5) choice of FM as a career assessed by five items. Perception aspects were assessed on a Likert scale with 5 points as ‘Strongly agree’, ‘Agree’, ‘Neutral’, ‘Disagree’ and ‘Strongly Disagree’. Items assessed in each part on the Likert scale are shown in Box 1. Closed-ended questions were used to capture participants’ data, and semi-structured questions were used to capture avenues of interaction with FM physicians and reasons for not choosing FM as a speciality. The questionnaire was created on Microsoft^®^ Forms.

BOX 1 Study questionnaire – Items assessed on a Likert scale.

1. **Choice of medicine as a career**
  - Medical School was my first choice
  - I chose medicine because it will provide me with financial well-being
  - I chose medicine because of the prestige it will bring me
  - I chose medicine because of parental influence
2. **Perception of Family Medicine as a speciality**
  - I know the job description of a Family Medicine physician
  - I think that Family Medicine is not important
  - Family Medicine physicians provide healthcare to the majority of the population
  - Our first-year medical school curriculum advanced my understanding of Family Medicine
  - My first-year experience has influenced my interest in pursuing a career in Family Medicine
3. **Choice of Family Medicine as a future career**
  - I have already thought of my future medical specialisation
  - I would be unhappy with Family Medicine as a career
  - Family Medicine will fulfil me in my career

### Survey distribution and data collection

The survey tool (Figure 1-A1) was self-administered. An introductory email providing information about the study’s significance and inviting participants was sent to all first-year MBChB students through their shared first-year group email address. A Microsoft^®^ link to the survey questionnaire was shared through the same email address. Weekly email reminders were also sent during the 4-week data collection period.

### Data analysis

The data collected were automatically captured on Microsoft^®^ Excel^TM^. It was cleaned, analysed and visualised using Microsoft^®^ Excel™ and STATA^®^ version 15 (StataCorp LLC^®^, United States). Frequencies and percentages for categorical variables and continuous variables were calculated and summarised as mean ± standard deviation (s.d.). Because of this survey’s pre-determined small sample size, the study was not powered to conduct a conclusive bivariate analysis of the collected data.

### Ethical considerations

Ethical approval was sought and obtained from the AKU Institutional Scientific and Ethics Committee (ISERC No. 2024/ISERC-87 [v3]), and a research permit was obtained from the National Commission for Science, Technology and Innovation (NACOSTI), Kenya. Informed consent was sought from all first-year students prior to their participation through an informed consent form shared electronically with all invited participants. To ensure confidentiality, no participant’s names or other identifying information were collected, and a unique code was automatically assigned once a survey response was submitted.

## Results

### Participants’ characteristics

Of the 59 first-year MBChB students at AKU, Kenya, 49 participated in the study, yielding an 83% response rate. The mean age of the participants was 19.4 years (s.d. 1.04), with the male-to-female ratio nearly equal (49% and 51%, respectively). Most respondents (82%) went through the 8-4-4 high school curriculum in public or private high schools prior to joining the MBChB programme. This is a Kenyan education curriculum where students went through 8 years and 4 years of primary and secondary school, respectively, prior to joining university. The other respondents (18%) progressed through international education curricula offered in Kenya.

### Perception towards Family Medicine as a speciality

When asked about their future career choice, only one participant indicated that they would choose FM. Eighteen (approximately 37%) of respondents indicated they would not choose FM, while 30 (approximately 61%) were uncertain. Five key points assessed the perception of FM as a speciality among the participants. These included knowledge of FM physician job description, the importance of FM practice and the influence of the first-year curriculum in understanding FM. Figure 1 shows the distribution of responses across different perception areas and indicates the contrasting perspectives. The values on the right (blue region) and those on the left (orange region) represent the frequency of those who responded in agreement or disagreement, respectively, with the five points as evaluated. The values in the middle (grey region) indicate the total number of neutral responses.

**FIGURE 1:**
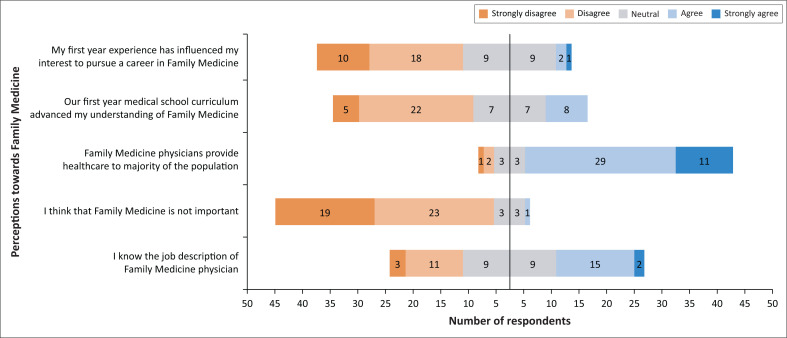
Distribution of responses across different perception areas of Family Medicine showing contrasting perspectives.

The survey’s findings also showed that during their first year, students engaged with FM physicians through channels that included tutorials (30 responses), guest lectures (20 responses), mentorship programmes (10 responses) and through other administrative roles held by FM physicians (32 responses). Other interaction channels with FM physicians cited included a simulation conference or as a patient at the Aga Khan University Hospital, Nairobi.

**FIGURE 2:**
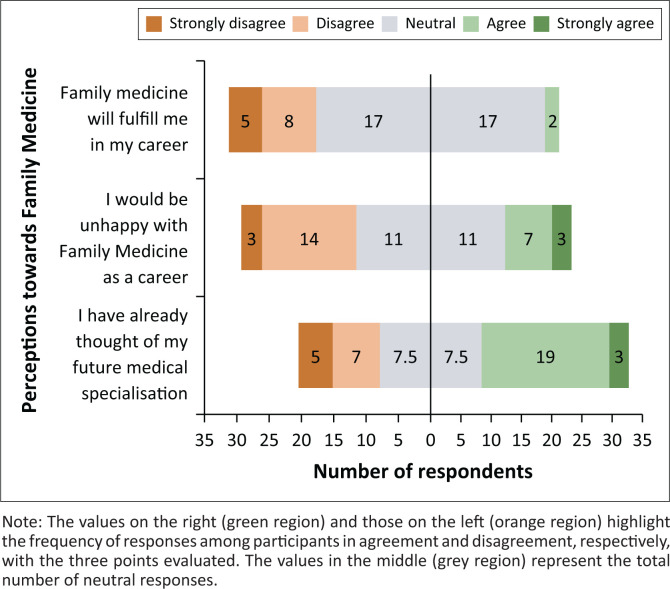
Distribution of responses across varied perspectives of choice of Family Medicine as a future career.

Perceived fulfilment in FM as a future career was assessed using three points. Figure 2 shows the distribution of the participants’ responses, indicating contrasting perspectives. The values on the right (green region) and those on the left (orange region) highlight the frequency of responses of participants in agreement or disagreement, respectively, of the three points evaluated. The values in the middle (grey region) represent the total number of neutral responses.

The respondents who indicated that they would not choose FM as a future career cited various reasons for this, including having no interest in FM (14 responses) and not knowing much about the discipline (5 responses). Respondents indicated four other reasons for their unwillingness to choose FM: (1) Previous negative interaction with FM physicians; (2) low financial incentives; (3) limited advanced training opportunities; and (4) poor systems to support FM practice.

Some of the responses are quoted as follows:

‘I have no interest, low financial incentive, underappreciated, overworked. In terms of fellowships, not as many, and the opportunities are greater for Internal medicine [*cardiology, neurology, nephrology, amongst many others*] and surgery [*many options*].’ (Respondent #07)

‘I have had very negative experiences with family medicine specialists, as they seem inauthentic.’ (Respondent #15)

Other medical students viewed FM with mixed feelings of positivity and indecision:

‘The course is very key in serving the roles of a doctor in the familial context. However, most family medicine specialists interact very negatively with many of the patients and in the medical training … this makes some residents acrimonious, and most think of them as inauthentic people. I hate apathy; I want to work with people I can converse with. All in all, family medicine is a good field.’ (Respondent #11)

‘I want to go into something surgical, but my thoughts on surgery may change as the years progress. However, for now, there is no speciality I dislike, and family medicine seems like a decent choice should I deviate from surgery in the future. In Kenya, a family medicine physician and a General Physician [*M.O*.] are more or less the same. As a country, we don’t have the systems or the cultures to support family medicine.’ (Respondent #29)

**FIGURE 3:**
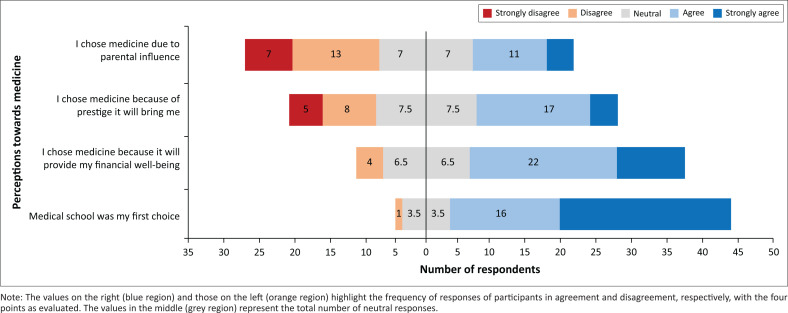
Distribution of responses across varied perspectives of medicine as a career choice.

In addition, 41 (84%) respondents indicated that medicine was their first choice for study, only one respondent indicated that it was not, while seven (14%) had a neutral response. On examining factors that influenced their choice of studying medicine, 32 (65%) strongly agreed or agreed that the prospect of financial well-being influences them, while 21 (approximately 43%) and 15 (approximately 31%) strongly agreed or agreed that prestige and parental influence, respectively, were their main drivers (Figure 3).

## Discussion

Despite many of the first-year students surveyed recognising the importance of FM, most did not fully understand its scope or the specific duties of the family physician. This echoes an observation from a previous study in Kenya, which showed a significant lack of clarity on how FM should be implemented in practice, despite FM having been defined by the Kenyan Ministry of Health.^8^ This study showed that at least 37% of respondents would not choose FM as a career in the future. This could be because of an overall paucity of understanding about this speciality and its practice in Kenya. Such low interest has also been reported in other studies in Africa. For instance, a study that reviewed career choices among final-year medical students from six sub-Saharan African countries showed that only 4.5% of the participants were interested in FM.^18^ Similarly, a study in South Africa observed that only 9% to 23% of students were interested in pursuing FM as a career,^14,15^ and another showed that FM was ranked as the sixth most preferred specialisation choice^16^ among final-year medical students. In Ghana, one study indicated that despite 88% of undergraduate medical students being aware of FM as a speciality, only 2.4% considered pursuing FM for postgraduate training, mainly because of inadequate understanding of the speciality.^12^ In contrast, a study conducted in Nigeria among medical students in their clinical years showed that FM was the second most popular speciality after surgery.^17^ All respondents felt that FM was relevant, 68.9% understood FM well, and 57.3% perceived it positively as a career speciality. It is worth noting that FM training in Nigeria started more than 50 years ago, a factor that would lead to the high interest indicated in that study.

Compared to these findings, our study focused uniquely on first-year students – a group for whom early perceptions may be more malleable. However, data on first-year medical students’ attitudes towards FM in Africa remains scarce. Most existing studies, as observed, focus on final-year cohorts, whose views are likely influenced by cumulative clinical experiences and prevailing institutional cultures. This gap underscores the value of our study in capturing baseline perceptions at the beginning of medical training.

Interaction with practising FM physicians is one potential avenue to augment the understanding of FM as a speciality. Here, we showed that the first-year students had multiple interactions with family physicians through lectures, tutorials and mentorship programmes. However, there was still a general lack of understanding of what FM entails. This could in part explain why very few students are interested in pursuing a career in this speciality. It has previously been shown that informal one-on-one career guidance, such as with personal tutors or seniors during placements, is more effective than formal career advice.^19^ Such an approach for guiding undergraduate medical students would help to bridge the gap in the knowledge, training and practice of FM in Kenya and the region.

This survey showed some negative sentiments from participants towards the FM physicians they had previously interacted with; at least two participants mentioned that family physicians seemed “inauthentic”. Career decisions are often honed over multiple experiences and potentially numerous years; one negative experience can lead to ruling out a career path.^19^ As such, negative experiences could be another contributing factor to a low number of respondents indicating interest in pursuing a career in FM. In our study’s context, the first-year students surveyed had not yet been attached to FM clinics but had interacted with FM physicians in other settings, for instance as patients. Curricula changes could address this gap by introducing early longitudinal exposure to FM practice and clinics. Indeed, longer placements in a specific field have been shown to increase the authenticity of an experience.^19^ It is also shown that having a role model can significantly impact a student’s career path.^19,20,21^ Taken together, increased contact time between medical students and practising family physicians may help to create additional avenues for role-modelling and mentorship.

Given that FM is a relatively new speciality in Kenya, there is a paucity of robust support systems for its training and practice despite some indicated growth.^9,10^ Our study suggests that this is an area of concern among students, some of whom feel that there are no systems in place to support FM, and that a career in this area would be less rewarding. The limited exposure to family physicians in the preclinical years of undergraduate medical programmes largely impacts student’s interest, an aspect also found in Canadian medical schools.^21^

Interestingly, 69% of our cohort were unsure whether a career in FM would offer fulfilment. Although limited research exists on what brings fulfilment in a FM career, studies in broader healthcare contexts have identified factors such as independence,^22^ new skills^23^ and altruism,^24^ as influencers of a healthcare professional’s sense of satisfaction. These attributes could feasibly be incorporated into a career in FM. Furthermore, some personal aspects of quality of life known to increase the happiness of healthcare professionals, such as taking breaks away from work, undertaking recreational activities, spending quality time with family and friends and getting good quality sleep,^22,25,26,27,28^ might be challenging to realise especially in the absence of adequate support. This is a point of concern among medical students and an area for further research to evaluate indicators of fulfilment in a career as a family physician in our context

Arguably, career preferences may not remain stable throughout one’s medical school training.^29,30^ Therefore, research involving the same cohort of students later in their undergraduate medical training would be insightful, given that 55% of the cohort reported not seriously considering their career path yet. In addition, research among students across different medical schools in our region would provide a wholesome and generalisable picture of the perception of FM among undergraduates.

### Limitations

Firstly, our study was performed at a single medical training institution; as such, the results may not be representative of all first-year medical students in the country. Inclusion of all higher education institutions approved to offer undergraduate medical education in future studies would allow generalisability of findings. Secondly, while the study explored reasons for disinterest in pursuing FM as a future career, it did not probe into the specific motivations behind interest or the uncertainty expressed by some participants. This information would be useful to identify additional factors, which influence students’ attitudes towards FM. Finally, in general, first-year medical students are not adequately exposed to all possible areas of specialisation in medical practice, including exposure to FM, as evidenced by the many neutral responses. There is therefore a need for a longitudinal assessment of evolving perceptions among medical students as they advance through their training.

## Conclusion and recommendations

A minority of first-year medical students surveyed were interested in pursuing FM as a career, with many respondents disagreeing that the curriculum advanced their understanding of the speciality. We recommend that medical schools better integrate FM training into their undergraduate medical curricula, as well as offer longitudinal exposure to FM, therefore foster understanding and a sense of authenticity among students. Furthermore, the inclusion of medical educators’ perspectives would provide insight into any curriculum gaps and offer opportunities to strengthen FM student exposure and practice. In a wider context, advocacy to increase awareness and scope of FM will be beneficial in attracting the necessary support for its training and practice in our region.

## Figures and Tables

**FIGURE 1-A1:**
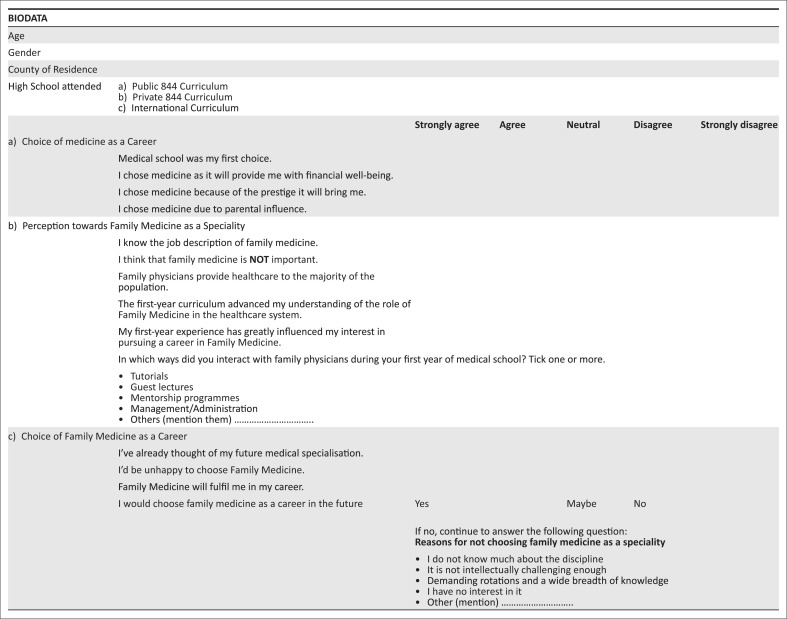
Questionnaire.
